# Supplementary material for: Sustainability of facilities built under the Community-Led Total Sanitation (CLTS) implementation: Moving from basic to safe facilities on the sanitation ladder
Source: PLoS One. 2023 Nov 16;18(11):e0293395. doi: 10.1371/journal.pone.0293395 (PMC10653469; doi:10.1371/journal.pone.0293395)
Supplement: S1 File — (DOCX) [file pone.0293395.s001.docx]

**Interview guide for the NGO APS, the Ministry in charge of Water and Sanitation and the National Institute of Statistics and Demography**

1. Date of interview: ....................................................................................

| **Simplified respondent consent form** |
| --- |
| My name is _______ [name of investigator] and I am working on a survey on the impact of Community-Led Total Sanitation (CLTS) in the Sissili province on sanitation improvement. The study is being carried out by the International Institute for Water and Environmental Engineering (2iE) in Ouagadougou, Burkina Faso. We would like to know if there have been any changes in hygiene and sanitation in these communities since the implementation of the CLTS approach in these villages. We assure you that any personal information you provide will remain confidential. The outcomes of this interview will be used for academic and scientific purposes only. Do you have any questions before we start? |

1. Do you agree to participate in this survey? (if NO, end the survey)

**•** Yes **•** No

1. Would you allow us to record this interview with our Dictaphone?

**•** Yes **•** No

1. Name and surname of respondent:
2. Position of respondent:
3. Phone number of the respondent:
4. Department / service:
5. How many villages are there in Sissili?
6. How many Open Defecation-Free (ODF) villages are there in Sissili?
7. What is the population size of the various municipalities in Sissili?
8. What is the number of households in the different municipalities of Sissili?
9. What was the number of latrines in Sissili before the CLTS was implemented?
10. What was the number of latrines in Sissili after implementation of the CLTS?
11. Can you provide us with the dates of ODF certification for villages in Sissili?
12. What was the rate of access to sanitation in Sissili before CLTS?
13. What was the rate of access to sanitation in Sissili after the CLTS?
14. How do you calculate sanitation coverage and access rates?
15. Can you provide us with your annual activity reports from 2016 to 2020 in Sissili?
16. What factors do you think have contributed to the evolution (reduction, increase or stagnation) of sanitation access rates in Sissili?
17. What do you think of the facilities provided by households as part of the implementation of the CLTS?
18. If you had to improve one thing in the CLTS approach, what would you change?
19. In your opinion, is CLTS the best approach to end open defecation, increase sanitation access and achieve SDG 6.2?
