# Supplementary material for: Sustainability of facilities built under the Community-Led Total Sanitation (CLTS) implementation: Moving from basic to safe facilities on the sanitation ladder
Source: PLoS One. 2023 Nov 16;18(11):e0293395. doi: 10.1371/journal.pone.0293395 (PMC10653469; doi:10.1371/journal.pone.0293395)
Supplement: S2 File — (DOCX) [file pone.0293395.s002.docx]

**Questionnaire for households Surveys in Open Defecation-Free (ODF) Communities in Sissili Province**

**Section A - General Information**

1. Survey number:
2. Date:
3. Start time:
4. Name of the village:
5. Name of the province:

| **Simplified respondent consent form** |
| --- |
| My name is _______ [name of investigator] and I am working on a survey on hygiene and sanitation. It is a study carried out by the International Institute for Water and Environmental Engineering (2iE) which aims to investigate sanitation facilities in your province, more specifically latrines. We would like to know whether you have latrines, in what context you obtained or built them, the type of latrine, the reasons for this choice and the durability of the latrine. So, we would like to ask you a few questions about this. The survey will last around 20 minutes. We assure you that any personal information you give us will remain confidential and anonymous. This is a voluntary survey, and you are free not to answer every question and/or to stop the survey at any time during the questionnaire. Nevertheless, we hope you will participate, as your opinion is important to us. It is also important to understand that we are only here to find out about the living conditions of the local population, and not to provide a humanitarian response such as donations or money. Whether or not you are surveyed has no bearing on whether or not you will be individually selected to receive assistance at a later date. The results of the survey will be used for academic and scientific purposes only. Do you have any questions before we start? |

1. Do you agree to participate in this survey? (if NO, end the survey)

**•** Yes **•** No

1. Gender

**•** Male **•** Female

1. Age of respondent:

(*********The respondent cannot be under the age of 18. If this is the case, thank the person, end the survey but save the form anyway*.**)**

1. Professional activity of head of household:
2. Household size:
3. Number of men in household:
4. Number of women in household:
5. Number of children under 5 years old in the household:
6. Does your household have a latrine?

**•** Yes **•** No

1. If yes, how many latrines does the household have?
2. How long have you had this (theses) latrine (s)?

**•** Before CLTS **•** After CLTS

**Section B - Latrine Status and Data**

1. If YES to question 14, can you show me?

**•** Yes **•** No

1. What type of latrine (interviewer's observation)?

- Unimproved pit latrine
- Sanplat
- VIP
- Ecosan
- Flush toilet
- Other, please specify:

1. Is there any evidence that the latrine is being used? (interviewer's observation)

• Yes • No

If YES, which one(s)?

1. What is the defecation slab made of? (interviewer's observation)

- Concrete
- Wooden
- Bamboo
- Masonry
- Terracotta
- No slab
- Other, please specify

1. Is there a cover for the defecation hole? (interviewer's observation)

• Yes • No

1. Is the lid on the hole? (interviewer's observation)

• Yes • No

1. What are the walls of the latrine made of? (interviewer's observation)

- Wood
- Bamboo
- Masonry
- Sheet metal
- Fabric
- No walls
- Other, please specify

1. What is the roof of the latrine made of? (interviewer's observation)

- Stubble
- Sheet metal
- Plastic
- Without roof
- Other, please specify

1. Is there a vent pipe? (interviewer's observation)

• Yes • No

1. What is the latrine door made of? (interviewer's observation)

- Wood
- Bamboo
- Sheet metal
- Steel
- Fabric
- Without door
- Other, please specify

1. Where do household members defecate when they go to the fields or when they are away from home?

- Bush/open defecation
- Cat method
- Public latrine/school/clinic
- Household latrine
- Other, please specify

1. Do all household members use the latrine for defecation?

• Yes totally (always) • No partially • I do not know

1. Does the latrine have any of the following problems?

- Collapse of the soil/slab/superstructure
- High water table rising through the bottom of the pit (especially in the rainy season)
- Subject to flooding during the rainy season
- Rapid filling of pit
- Other (please specify)

1. In the past 12 months, have you performed any repairs or maintenance for any of the following?

• Yes • No • I do not know

If YES, what problems?

- Collapse of the slab or superstructure
- Roof problems
- Slab problems
- Pit overflow
- No water in tank
- Pipe failure
- Other (please specify):

1. Do collapses and problems with the latrine recur?

• Yes • No • I do not know

1. At what time of year do these collapses and latrine problems usually occur??

• Rainy season • Dry season

1. Reasons for choosing the type of latrine built by the household?
2. Would you be willing to invest more money to get an improved latrine (if not)?
3. Did a household member attend the CLTS triggering?

• Yes • No

**Section C – ADDITIONAL INFORMATION**

1. Comments or observations
2. End time:
3. GPS coordinates:
