# Supplementary material for: Sustainability of facilities built under the Community-Led Total Sanitation (CLTS) implementation: Moving from basic to safe facilities on the sanitation ladder
Source: PLoS One. 2023 Nov 16;18(11):e0293395. doi: 10.1371/journal.pone.0293395 (PMC10653469; doi:10.1371/journal.pone.0293395)
Supplement: Attachment — Submitted filename: Review comments_sustainability of facilities by a CLTS intervention.docx [file pone.0293395.s003.docx]

Overall, this study provides a very insightful messeages to the global sanitation community. Indeed it has great policy and programmatic implications that need to be incorporated into sanitation policy and also programmes surrounding sanitation and community-led total sanitation. However, I hope that the could refine the manuscript, particularly the methods part. Please refer to the following comments and suggestions.

Can they rephrase their study aim, objectives and hypothesis in more neutral way? The current description regarding the study aim, objectives and hypothesis are described in a sort of biased way. For instance, they stated that “this paper seeks to provide evidence that the latrine technology used in CLTS does not increase sanitation access, and propose a few suggestions…”, and also “the objective of this study is to confirm that the latrine technology used in CLTS does not lead to sanitation access, and thus propose the ways to promote…”. As a research paper, I would suggest that the authors rephrase the study objectives in more neutral way, for instance: “we aim to assess whether the latrine technology used in CLTS did increase….in rural villages of Burkina Faso”, and I also suggest that they delete this, “and propose a few suggestions…” since this is not a policy paper and they could include their suggestions in the discussion part not in the results part.

Can the authors move all the paragraphs of 1 (intervention of the CLTS approach in Burkina Faso) to 5 (data analysis) to Methods part?

**(3. Methods and tools)**

The first paragraph is explaining study objectives, which should be moved to the introduction.

As for the data collected from the NGO (APS), more explanations should be added including: who conducted the surveys; when did they conduct; how much size and what methods they applied; validity and reliability of the surveys; and the like.

**(sample size calculation formula)**

The authors cited this “The non-response rate is considered acceptable when it is less than 10% [37]”. However, when designing sample size, it would be better to estimate the non-response rate in a conservative manner so that they could sample as large as possible. I guess that 5% of non-response is too small. I would suggest that they describe somewhere in the results part about the actual non-response rate in their survey.

The WHO manual for sample size estimation explains different formula for different situations and it would be better if authors could explain the rationale why they used the particular formula. In addition, I hope they could rephrase the explanations about the P in the formula. I guess that P in the formula could be redescribed as latrine coverage instead of maintaining current description. If that was true, they also have to explain why they assumed that the latrine coverage was 50% (meaning P=0.5). In fact, they explained that 7 villages were selected in page 8, which means that they seemed to have applied “two-stage sampling methods”; however, the sampling formula they used were based on simple random sampling.

Can they explain how those 7 villages were selected and what particular sampling methods were used? Based on their explanation, they seemed to have used purposive sampling. If that was the case, next question is why they decided to sample “seven (7)” villages, which is related to sample size calculation. In page 9, they stated that they used “the probability proportionate to size” methods, which is called PPS sampling. The PPS sampling is used when selecting clusters (which means village in this paper) not households or individuals. In the same paragaph (the first paragraph in page 9), they explained how they calculated the number of households and this are not related to the PPS sampling. Normally for the PPS sampling, the sample size per village (cluster) is same regardless of the village population because the probability of selecting a village of larger population became higher than the smaller villages. I guess they allocated sample size per village in proportionate to the village population. I hope they could rephrase all the paragraphs related to sampling, and explain the limitations of the sampling methods somewhere in the discussion part.

Please add the table number in page 8, and also “,” should be changed to “.”. Alternatively, I would suggest that they delete this table since it is commonly accepted table in basic statistics, and all the values in the table were not developed by the authors.

“Oral informed consent…”.

- This sentence should be moved to the part of Ethical considerations.

Can they provide the questionnaire set as a supplementary material?

“The household surveys were conducted during the rainy season in two campaigns.”

- Does this mean that they conducted the one-time survey for two years (2021 and 2022)? Or did they conduct two rounds of survey targeting 410 households? If the first was true, how could they explain the one year difference between the two surveys? Why did the need 2 years for conducting relatively small scale survey?

They should describe in more detail regarding the respondents (stakeholders) other than households in the Table 1 since most of the explanations in page 8-10 were only about the households sampling. For example, why and who interviewed the NGO implementers, institutional/state actors, community stakeholders. Also, please provide interview guide or questionnaire (even it happened to be unstructured) as a supplementary material.

4. ethical consideration

Can they provide more explanations about the ethical approval from the ethical committee or ethical review board including the approval number?

5. data analysis

“as recommended by [41]”

Can they repharase this by adding the scholars’ names to the sentence instead of just putting reference number?

Having read all the manuscript, I think theis study is quantitative rather than qualitative study. The majority of the results are based on quantitative analysis. I would suggest that the authors either delete explanations about qualitative methods and their results or add more explanations on methods and the results about the qualitative analysis both in methods and results part.

6. Result

Introduction, Methods, Results and Discussion are the same level of heading, and thus number 6 is not appropriate for the Results part.

The paragraph includes explanations on “methods”. Can they explain some part of this paragraph also in the methods while maintaining this paragraph in the results as they are?

I would suggest that they maintain the Table 3 in the main body of the manuscript and attach the Table 2 as a supplementary material for simplicity instead of presenting the two tables in the main body.

Can they explain more details about the Table 2 and 3 in methods? I would expect that the authors will describe details about the 7 surveyed villages, but they explained much details about the coverage in other villages in the Results, also the number of villages was also 7. I hope they could exlain more about the methods and data they got from APS & INSD in the methods part. In additin, add some explanations as a footnote for the Table 3.

In table 5, Can they add % in addition to the number?

In page 23, can they delete “the sad fact” since it is subjective expression.

The all paragraphs starting from the “This low….” in page 24 should be moved to the discussion part since it is not results.

Discussion

In the first paragraph of the discussion in page 26, “to some information” should be deleted or rephrased.

Can they include the limitations of this study in the discussion part?
