# Supplementary material for: Sustainability of facilities built under the Community-Led Total Sanitation (CLTS) implementation: Moving from basic to safe facilities on the sanitation ladder
Source: PLoS One. 2023 Nov 16;18(11):e0293395. doi: 10.1371/journal.pone.0293395 (PMC10653469; doi:10.1371/journal.pone.0293395)
Supplement: Attachment — Submitted filename: PONE-D-23-13662_review.docx [file pone.0293395.s004.docx]

Review of Kouassi et al, Sustainability of facilities built under the Community-Led Total Sanitation (CLTS) implementation: Moving from basic to safe facilities on the sanitation ladder

PONE-D-23-13662

Summary: This manuscript presents results of interviews conducted over two consecutive rainy seasons with households and key informants associated with a deployment of community-led total sanitation (CLTS) in 7 villages of Sissili Province, Burkina Faso, to assess the program performance with respect to the objective of sustained increases in household sanitation coverage. It also draws on latrine statistics provided by an NGO that implemented CLTS in Sissili (the Association for Peace and Solidarity – APS) as well as government authorities. It concludes that while CLTS is has resulted in a vast increase in household sanitation “coverage” (defined in Burkina Faso as a latrine serving no more than 10 individuals), durability of latrines installed during CLTS is poor, threatening the sustainability of these coverage gains, and leading to a much more modest effect on sanitation “access” (defined by the government of Burkina Faso as the presence of “basic sanitation” – presumably per the WHO/UNICEF JMP definition).

Broad reflection: This manuscript offers important reinforcing evidence of the shortcomings of CLTS that have been raised elsewhere but which it does not cite (see for example: Trimmer, J et al. 2022. The Impact of Pro-Poor Sanitation Subsidies in Open Defecation-Free Communities: A Randomized, Controlled Trial in Rural Ghana. Environmental Health Perspectives. DOI:10.1289/EHP10443; Brown, J., et al. 2019. Community-Led Total Sanitation Moves the Needle on Ending Open Defecation in Zambia. American Journal of Tropical Medicine and Hygiene 100 (4) 767-769. DOI:10.4269/ajtmh.19-0151; and Cameron, L., et al. 2021 Sanitation, financial incentives and health spillovers: A cluster randomised trial. Journal of Health Economics. DOI:10.1016/j.jhealeco.2021.102456). Its interviews with households over two seasons along with field observations of the sanitation infrastructure are important, particularly as they lend themselves to the sobering recognition of the durability problem as perhaps the most critical shortcoming of CLTS. The authors’ questions about the effectiveness of CLTS’ zero-subsidy principles are also critical to understanding CLTS’ slippage issues.

In its current state, however, this manuscript is unpublishable in a peer-reviewed venue. Most problematically, its stated objectives are inconsistent with the core scientific convention of posing neutral questions which are either answered (or not) by the evidence collected (see page 7 of the manuscript). Determining that
“CLTS increased coverage,” or that “the quality of latrines built under CLTS is poor” are statements of results, not neutral research objectives; indeed, framed as research objectives, they reveal bias. Instead, the objectives should be framed as questions, e.g. “how did CLTS change sanitation coverage in Sissili Province?” or “what is the failure rate of latrines installed under the CLTS program in Sissili Province?” or “what explains the high failure rate of latrines installed under CLTS in Sissili Province?”

There are other significant issues. For example, the authors report two waves of data collection, but do not report results from each wave, nor do they do indicate how the two waves of data collection differed. From what I could tell, they report the two waves of household surveys as though it were a single cross-sectional survey collected during a single survey period, but it’s not clear.

Also: much of the results section presents data unrelated to the authors’ own household survey results. Tables 2 and 3 and Figure 8 all correspond to “corrected” self-reported latrine coverage data from the NGO APS.

Meanwhile, the objectives of the power calculations for the household surveys are not obvious to me. Typically power calcs are designed to communicate the smallest detectable difference between an intervention group and a counter-factual group with respect to a specific variable of interest in a controlled trial, or else, in the case of a quasi-experimental study, to explain the variation in a response variable with respect to the variation of one or more explanatory variables, or to determine the sample’s “representativeness” of a broader population. None of that is offered here. At best, this is an effort to collect some “ground truth” on CLTS performance in Sissily Province that may be illustrative of broader trends but not conclusive.

Also: the authors make claims about how CLTS has affected open defecation in Sissily province (they use the term “cessation of OD”) but they neither measure OD themselves nor refer to any other studies of open defecation (as distinct from latrine coverage or “access.”)

Also: the recommendations regarding CLTS are intriguing, but they don’t necessarily follow directly from the data presented or directly collected by the authors, and are better suited for some kind of commentary or policy analysis piece, rather than a research article as presented here.

In short, I very much hope that a sensible venue and format can be found for the team’s findings of latrine durability challenges and its associated drivers related to affordability of durable materials and professional latrine installation, but I don’t see an academic journal research article as offering them.
