# Supplementary material for: Sustainability of facilities built under the Community-Led Total Sanitation (CLTS) implementation: Moving from basic to safe facilities on the sanitation ladder
Source: PLoS One. 2023 Nov 16;18(11):e0293395. doi: 10.1371/journal.pone.0293395 (PMC10653469; doi:10.1371/journal.pone.0293395)
Supplement: Attachment — Submitted filename: Response to_Reviewers&editor.docx [file pone.0293395.s005.docx]

# Responses to questions, comments and suggestions from editor and reviewers

We, the authors, would like to thank the editor and reviewers for the time that you have spent on our manuscript, for your valuable comments, questions and concerns. We took into consideration, carefully, the proposed changes and they have been reflected upon and integrated into this revised version. For all questions and comments, please find below a point by point response, as well as background material and citations to new references, added to the new version of the manuscript. Whenever we felt it was necessary to explore the issue further, we provided a few changes to the manuscript for improvement. The comments are in blue and the responses from the authors are in black. We do hope this new submission, with the responses given below, will address the concerns raised by the initial manuscript.

**Editor #:**

- Your submission has some merit, but needs many improvements. So, I decided to give you a chance to improve it.

**Author’ response**: Thank you for your comment.

- Please shorten your abstract to max 250 words.

**Author’ response**: Thank you for your comment. However, according to the journal's guide of authors the abstract should not exceed 300 words not 250. The new abstract proposed in the revised manuscript is 284 words. Thank you, dear editor.

- You have very long paragraphs in the introduction, method, etc. Please use a maximum of 5-6 sentences (but also not very long) per paragraph. improve it.

**Author’ response**: Thank you for your remark. The authors have respected the limit of 5 to 6 sentences per paragraph in the revised manuscript as suggested.

- I don’t see any knowledge gaps proposed in this study. You only mention the objective without telling what is new in your study.

**Author’ response**: Thank you for your remark.

**Gap**: Most previous studies of CLTS have focused on its effectiveness in eradicating open defecation. CLTS does not subsidize sanitation facilities, letting households build their own facilities. The types and sustainability of facilities when construction is entrusted to households without guidelines and the contribution of CLTS in achieving SDG6.2, remain understudied. Our paper paper addresses these gaps

**New**: CLTS increases the latrine coverage rate but does not improve the sanitation access rate, as the facilities (latrine types) it generates are generally unimproved, not durable, not adequate, not safe for the user and for these reasons are not taken into account in the calculation of the sanitation access rate.

In Burkina Faso, a household has access to sanitation from a latrine in the "Limited", "Basic" or "Safely managed" categories of the sanitation ladder, which is not the case for the majority of latrines built by households as part of CLTS implementation.

The result is poor-quality, unsustainable technologies (latrines) that often put users at risk, as they regularly collapse during the rainy season and end up discouraging the least resilient households, who revert to the practice of open defecation.

According to SDG 6.2 (By 2030, ensure access to adequate and equitable sanitation and hygiene for all, and end open defecation), this document reveals that CLTS contributes to part of this goal by ending open defecation, but does not offer "adequate" access to sanitation. Adequate access means having a latrine that meets national or international standards, ensures the safety of users, is resistant and limits the risk of feco-oral contamination, which is not always the case with the majority of latrines built.

Finally, the paper points to the need for further research into social approaches such as CLTS, involving technical and engineering aspects, because beyond the socio-economic aspects, the sustainability of CLTS and the achievement of access to adequate and safe sanitation also depend on the durability and resilience of the technologies put in place.

- I agree with the 2nd reviewer, your objective is not neutral. It seems that you want to prove the assumption that you describe previously. Even, I don’t see a strong argument in the previous paragraph about your assumption, i.e., the latrine technology used in CLTS doesn’t lead to improved sanitation access. Also, as a WASH practitioner, I’m a bit confused by your objective because all types of sanitation technology (Pit latrine, VIP, etc.) increase sanitation access.

**Author’ response**: Thank you for your comment. A new, more neutral formulation of the paper's objectives has been made in the introduction section of the revised manuscript. You will find it on page 5 of the revised manuscript.

In Burkina Faso, as in many other countries, a household or community has access to sanitation if it has at least one latrine meeting minimum standards set at national or international level. According to the regulations in Burkina Faso, a household has access to sanitation if it has an improved latrine (VIP, SanPlat, flush toilet, at least a concrete slab, a masonry pit, an aeration pipe, a cover to close the hole and a roof, etc.) or any other type of latrine that ensures safety for the user with a ventilation pipe, a concrete slab and limits the risk of fecal-oral contamination to a maximum. In other words, if it has a latrine in one of the “Limited”, “Basic” or “Safely managed” categories of the sanitation ladder. The type of pit latrine that was widely used by households during the CLTS in Sissili does not meet all of these criteria and is classified as “Unimproved”. Households with these latrines were not considered to have access to sanitation in Burkina Faso. The result of CLTS implementation is a large number of poor-quality latrines, generally assimilated to unimproved pit latrines (see photo below) built within the framework of the CLTS, which are unfortunately not taken into account in the calculation of the access rate, as they do not ensure the safety of the user and do not comply with the set standards.

So, even if this household has stopped open defecation and acquired an unimproved latrine during CLTS implementation, it cannot be included in the calculation of the sanitation access rate. If we generalize this to the community level, we arrive at an ODF status, but with several latrines that do not meet the standards, and are therefore not taken into account in the calculation of the sanitation access rate.

**photo**: *Type of latrines generally built under CLTS and not considered in the calculation of the sanitation access rate in Burkina Faso*.

But it can happen, for example, that a village of 50 households without CLTS, where open defecation is commonly practised, has a sanitation access rate equal to or even higher than a village of 50 ODF households (following CLTS implementation). In fact, if in the non-ODF village, 10 out of 50 households have improved/safe latrines, and in the ODF village, all 50 households have latrines but do not comply, the access rate of the non-ODF village will be higher than that of the ODF village.

We developed this a little in the paper (Fig. 7) because the province of Sissili is the only one declared ODF in Burkina Faso at present, but paradoxically its sanitation access rate is lower than many other provinces where open defecation is endemic and where CLTS has not been implemented, which should not have been the case if CLTS had led households to build latrines meeting a minimum of standards.

So, we do not entirely agree with the statement that “all types of sanitation technology (pit latrines, VIPs, etc.) improve access to sanitation”, but rather that “all types of technology improve latrine coverage”. Yes, CLTS can eradicate open defecation and increase latrine coverage (i.e. the number of latrines built), but it does not always increase the rate of access to sanitation.

- Please organize better the section's name and numbering. You have an introduction section but then have sections 1, 2, 3 (method and tools), etc. Very messy!

**Author’ response**: Thank you for your comment. Efforts have been made to improve the organization and numbering of sections following the guidelines of the journal template in the revised manuscript.

- I think the introduction section is too long. Maybe you can shorten it a bit because you also have another section about CLTS intervention in Burkina Faso. Or maybe you can put that part in the introduction, but need to shorten it a bit as well.

**Author’ response**: We thank the reviewer for this suggestion. As suggested, the introduction has been shortened while being more precise, concise and keeping the most important elements in the revised manuscript. The section on CLTS intervention in Burkina Faso has been combined with the introduction in the revised manuscript.

- Please check your storyline. You mention Sissili in section 1, but then explain later this area in section 2.

**Author’ response**: Thank you for your remark. The sections in the revised manuscript have been reorganized to fill this gap.

- Again, storyline. It is strange that you mention objectives in the method section.

**Author’ response**: Thank you for your remark. We have removed the objectives from the method section and only mentioned them in the introduction to the revised manuscript.

- please check the neatness of the writing, e.g., the spacing on page 7 method and tools section.

**Author’ response**: We thank the editor for this remark. Spacing has been standardized in the revised manuscript.

- I think you can put the detail of the sampling calculation in the supplementary (page 8).

**Author’ response**: We thank the reviewer for this suggestion. But we believe that sampling and related details are an important element in a quantitative study such as ours, and deserve to be preserved in the body of the manuscript.

- “increasing the latrine coverage rate from 29.51% in 2016 to 107.66% in 2020” -> more than 100%, also in table 2? Please explain this.

**Author’ response**: Thank you for your comment. Indeed, in some cases we may have latrine coverage rates higher than 100% in a region.

In Burkina Faso, “latrine coverage” means that a latrine is used for a maximum of 10 people. So if a household consists of 15 people, for it to be considered "latrine coverage", it must have 2 latrines. So, if for example, the population of a region is estimated at 100,000. To have 100% latrine coverage, it needs 100,000 / 10, i.e. 10,000 latrines. So, if 10,000 latrines are counted during the enumeration in this region, the coverage rate will be 100%, but if more than 10,000 latrines are counted, the coverage rate will be higher than 100%. However, a coverage rate of 100% or 110% does not mean that every household has a latrine, NO. An affluent household of 4 may have 2 or 3 latrines, while a household of 15 may not. the coverage rate can reveal internal disparities.

If the latrine coverage rate were calculated on the basis of the number of households, we could not have had rates of over 100%, but it is calculated on the basis of population size. This explains to some extent the rate of over 100%. But the calculation of the access rate takes into account types of facilities with precise specifications and is calculated on the basis of the number of households, unlike latrine coverage. That's the difference between the latrine coverage rate and the latrine access rate.

We have explained this better on page 6 of the revised manuscript.

- If you already have the graph and table, don’t need to explain in detail in the paragraph, e.g., page 14.

**Author’ response**: Thank you the reviewer for this suggestion. We have taken this into account in all the revised manuscript. Thank you again.

- The explanation about types of latrine may not be necessary unless the definition used in the study area is different than what we know/literature say. Or you can move it to the supplementary.

**Author’ response**: We thank the editor for this suggestion. The Sanplat and VIP types identified in the study area are the same as in the literature, so the explanation of these two types has been removed from the revised manuscript. As for the pit latrines, an explanation in the manuscript was necessary because they differ from what is found in the literature.

- Please edit the way you report the quotes in the journal. You must follow the “qualitative” approach here, see for example: <https://www.nature.com/articles/s41598-023-30586-z>.

**Author’ response**: Thank you for your comment. All the quotes in the revised manuscript have been put in the same format as the example on the referenced article.

- There are some paragraphs that consist of one sentence only. Please edit it.

**Author’ response**: Thank you for your comment. All paragraphs containing a sentence have been modified or combined with other paragraphs in the revised manuscript.

- I think the proposed guidelines may be located in the discussion section. Also, I never see any scientific journal describing step-by-step guidelines/procedures. I think you need to remove it or put it in the supplementary.

**Author’ response**: Thank you for your comment. With the other co-authors we have agreed that you are right on this point and have therefore decided to remove the section “Proposed guidelines for the construction of approved and sustainable latrines as part of the CLTS approach to reduce relapse and also increase sanitation access rates” in the revised manuscript.

- In the conclusion, you write “it does not guarantee a significant increase in the rate of access to sanitation”. Please remove the statistical word “significant” there.

**Author’ response**: Thank you for your comment. The word "significant" has been removed from the revised manuscript.

- You write that the main objective of your analysis is about latrine technology (in the introduction), but then your guidelines describe CLTS in general, not only latrine technology. I think you must change your main objective, otherwise focus your recommendation on the latrine technology and not general CLTS.

**Author’ response**: Thank you for your comment. The objectives of our paper were modified to fit with the results obtained and presented. After discussion with the co-authors we saw that the recommendations as presented made our paper look more like a policy document than a research paper. So, this part was removed from the revised manuscript and the emphasis was really on technologies as you suggested.

Thank you again for all your comments and time in reviewing our paper.

**Reviewer #1:**

- Overall, this study provides a very insightful messages to the global sanitation community. Indeed, it has great policy and programmatic implications that need to be incorporated into sanitation policy and also programmes surrounding sanitation and community-led total sanitation. However, I hope that the could refine the manuscript, particularly the methods part. Please refer to the following comments and suggestions.

**Author’ response**: Thank you for your comment.

- Can they rephrase their study aim, objectives and hypothesis in more neutral way? The current description regarding the study aim, objectives and hypothesis are described in a sort of biased way. For instance, they stated that “this paper seeks to provide evidence that the latrine technology used in CLTS does not increase sanitation access, and propose a few suggestions…”, and also “the objective of this study is to confirm that the latrine technology used in CLTS does not lead to sanitation access, and thus propose the ways to promote…”. As a research paper, I would suggest that the authors rephrase the study objectives in more neutral way, for instance: “we aim to assess whether the latrine technology used in CLTS did increase….in rural villages of Burkina Faso”, and I also suggest that they delete this, “and propose a few suggestions…” since this is not a policy paper and they could include their suggestions in the discussion part not in the results part.

**Author’ response**: Thank you for this pertinent comment and for your suggestion. Indeed, the editor has raised this point concerning the objectives of the paper. Based on your suggestion, a clearer and more neutral reformulation of the objective has been made. You can see it in the introduction section of the revised manuscript. Not being a policy paper and with the editor's recommendations, we have removed the “...propositions/ suggestions...” section in the revised manuscript in order to preserve its scientific aspect only.

- Can the authors move all the paragraphs of 1 (intervention of the CLTS approach in Burkina Faso) to 5 (data analysis) to Methods part?

**Author’ response**: We thank the reviewer for this suggestion. We have taken this into account in the new organization proposed in the revised manuscript, while also following the journal template. The former section (intervention of the CLTS approach in Burkina Faso) has been merged with the introduction in the revised manuscript. All the others have been put together in a section called "Materials and Methods" in the revised manuscript.

- (3. Methods and tools) The first paragraph is explaining study objectives, which should be moved to the introduction. As for the data collected from the NGO (APS), more explanations should be added including: who conducted the surveys; when did they conduct; how much size and what methods they applied; validity and reliability of the surveys; and the like.

**Author’ response**: Thank you for your comment. Thank you for your comment. We have taken this comment into account in the revised manuscript. The objectives has been moved to the introduction. Additional explanations concerning the data from the NGO have been added in the method section. We hope that the added information will answer your concern.

- (sample size calculation formula) The authors cited this “The non-response rate is considered acceptable when it is less than 10% [37]”. However, when designing sample size, it would be better to estimate the non-response rate in a conservative manner so that they could sample as large as possible. I guess that 5% of non-response is too small. I would suggest that they describe somewhere in the results part about the actual non-response rate in their survey. The WHO manual for sample size estimation explains different formula for different situations and it would be better if authors could explain the rationale why they used the particular formula. In addition, I hope they could rephrase the explanations about the P in the formula. I guess that P in the formula could be redescribed as latrine coverage instead of maintaining current description. If that was true, they also have to explain why they assumed that the latrine coverage was 50% (meaning P=0.5). In fact, they explained that 7 villages were selected in page 8, which means that they seemed to have applied “two-stage sampling methods”; however, the sampling formula they used were based on simple random sampling.

**Author’ response**: Thank you for your comment. Indeed, maximizing the non-response rate to 10% instead of 5% would have been judicious but it would also have increased our sample size. However, the authors have carried out surveys in this region in the past, where the majority of respondents provided answers, and almost no cases of non-response were recorded. As the authors' previous surveys in the region had never resulted in a response rate of more than 5%, the present study set the response rate at 5%. The characteristics of the respondents chosen (head of household, mother of the household, people over 18 years of age) who gave their consent also played a part in the fact that there were rarely any cases of non-response, all the more so as during the surveys we were accompanied by a member of the community, which reinforced the respondents' confidence. The rationale behind the choice of this formula has been added to the revised manuscript.

The choice of this formula is justified by 2 major elements: It maximizes a relatively larger sample size than that given by other formulas, notably the Cauchy formula, since it includes the non-response rate. As such, it also increases the reliability of the results obtained (1) and it is one of the most widely used formulas for sanitation studies. Several authors, such as Temesgen A et al, (2021); Koné et al, (2009); Dongo et al, (2008) used it to determine the sample size for health, sanitation and epidemiological surveys (2).

For the choice of P, we based ourselves on the guidelines of the sanitation memento (Gabert, J. (2018). *Mémento de l'assainissement* (p. 844). éditions Quae) (which generally defines it as 0.5 for sanitation studies) because before the study, we had no elements to fix it. P is expected proportion of a population response or actual proportion.

Yes, it was a two-stage sampling. We have clarified this point in the revised manuscript in the "methodology" section. The choice of the 7 villages was made on a reasoned basis according to certain criteria that we defined in the paper, notably the age of the village's ODF status, access, security, etc. But the choice of households within these 7 villages was random. So, the 410 households were surveyed on the basis of simple random sampling.

The justification for the choice of sampling formula, villages and households surveyed has been added to the "methodology" section of the revised report.

- Can they explain how those 7 villages were selected and what particular sampling methods were used? Based on their explanation, they seemed to have used purposive sampling. If that was the case, next question is why they decided to sample “seven (7)” villages, which is related to sample size calculation. In page 9, they stated that they used “the probability proportionate to size” methods, which is called PPS sampling. The PPS sampling is used when selecting clusters (which means village in this paper) not households or individuals. In the same paragaph (the first paragraph in page 9), they explained how they calculated the number of households and this are not related to the PPS sampling. Normally for the PPS sampling, the sample size per village (cluster) is same regardless of the village population because the probability of selecting a village of larger population became higher than the smaller villages. I guess they allocated sample size per village in proportionate to the village population. I hope they could rephrase all the paragraphs related to sampling, and explain the limitations of the sampling methods somewhere in the discussion part.

**Author’ response**: Thank you for your comment. In order to study the sustainability of the infrastructure built under the CLTS, the villages were selected on the basis of the age of their ODF status (the oldest in the zone were chosen), ease of access by vehicle and the risk of insecurity (as it has to be said, a large part of Burkina Faso is plagued by terrorism, which weakens the implementation of many projects in these areas). We then divided the calculated sample size proportionally to the population of the selected villages. A reformulation and further explanation of this part has been made in the revised manuscript. You will find it in the “Methodology” section of the revised manuscript.

- Please add the table number in page 8, and also “,” should be changed to “.”. Alternatively, I would suggest that they delete this table since it is commonly accepted table in basic statistics, and all the values in the table were not developed by the authors.

**Author’ response**: We thank the reviewer for this suggestion. We have removed this table from the revised manuscript as suggested.

- “Oral informed consent…”. This sentence should be moved to the part of Ethical considerations.

**Author’ response**: We thank the reviewer for this suggestion. The sentence has been moved to the Ethical considerations section as requested.

- Can they provide the questionnaire set as a supplementary material?

**Author’ response**: Thank you for your comment. The questionnaire used for the study were also added in supplementary material as requested.

- “The household surveys were conducted during the rainy season in two campaigns.”

Does this mean that they conducted the one-time survey for two years (2021 and 2022)? Or did they conduct two rounds of survey targeting 410 households? If the first was true, how could they explain the one-year difference between the two surveys? Why did the need 2 years for conducting relatively small-scale survey?

**Author’ response**: Thank you for your comment. The surveys were carried out only once in 2021 (during the rainy season) in 4 villages and only once in 2022 in 3 villages during the rainy season, which gives us two collection campaigns. It is the sum of households surveyed in 2021 and 2022 that has given the 410 obtained. According to the literature, latrines are not resistant to strong winds and heavy rains, so the period when these climatic elements come together in Burkina Faso is the rainy season. Burkina Faso is a Sahelian country with only one rainy season a year, which explains the choice of season.

The reasons why we were unable to survey all 410 households in a single year and did so over two years include:

- coup d'état in Burkina Faso during the first phase of data collection
- The reduced number of data collectors for the study;
- The budget allocated for the surveys for the minimum sample size calculated was relatively low, which necessitated a search for additional funding at the beginning of the following year, i.e. 2022;
- The unavailability of some field guides who understood the local language throughout the rainy season, as they had to attend to other duties. The majority of the population lives from agriculture, so during this period they are very busy in the fields to ensure their subsistence for the year, especially as there is only one rainy season in Burkina Faso;
- The long distances between villages;
- The unavailability of certain provincial executives to be in contact with village leaders who had to give us their prior agreement before the household surveys in their village;

- They should describe in more detail regarding the respondents (stakeholders) other than households in the Table 1 since most of the explanations in page 8-10 were only about the households sampling. For example, why and who interviewed the NGO implementers, institutional/state actors, community stakeholders. Also, please provide interview guide or questionnaire (even it happened to be unstructured) as a supplementary material.

**Author’ response**: Thank you for your comment. Details of other stakeholders besides households, and the information sought from them, have been added to the revised manuscript. For the example of NGO managers, it should be noted that any NGO which is to implement CLTS in a village must establish a baseline situation in order to know the number of latrines before the project, the number of households, the size of the village, etc. We sought to obtain this data from them, to compare it with that of the other stakeholders. We sought to obtain these data from them, compare them with those collected from institutional actors (Ministry in charge of sanitation, national statistics institute) and those declared by the households we surveyed. All this was done to ensure the reliability of the results presented and to avoid dependence on any one source of information.

The interview guides have been added as supplementary material to the revised manuscript.

- Ethical consideration: Can they provide more explanations about the ethical approval from the ethical committee or ethical review board including the approval number?

**Author’ response**: Thank you for your comment. Ethical approval details, ethical committee and approval number have been added to the “Ethical considerations” section of the revised manuscript.

- data analysis “as recommended by [41]” Can they rephrase this by adding the scholars’ names to the sentence instead of just putting reference number?

**Author’ response**: We thank the reviewer for this suggestion. We would have liked to add the scholars' name in place of the square brackets, but the journal's citation style does not allow it. So, we have adopted the citation style required by the journal.

- Having read all the manuscript, I think this study is quantitative rather than qualitative study. The majority of the results are based on quantitative analysis. I would suggest that the authors either delete explanations about qualitative methods and their results or add more explanations on methods and the results about the qualitative analysis both in methods and results part.

**Author’ response**: We thank the reviewer for this suggestion. As suggested, some of the qualitative elements (not all) have been removed and more details on the quantitative aspects have been provided in the revised manuscript both in the method section and in the results.

- Result Introduction, Methods, Results and Discussion are the same level of heading, and thus number 6 is not appropriate for the Results part.

The paragraph includes explanations on “methods”. Can they explain some part of this paragraph also in the methods while maintaining this paragraph in the results as they are?
I would suggest that they maintain the Table 3 in the main body of the manuscript and attach the Table 2 as a supplementary material for simplicity instead of presenting the two tables in the main body.

**Author’ response**: Thank you for your comment. The revised version of our paper has been reorganized according to the journal template, including the numbering of the sections. You can see this in the revised manuscript. We have taken your comment into account by deleting table 2 and keeping table 3 in the body of the revised manuscript.

- Can they explain more details about the Table 2 and 3 in methods? I would expect that the authors will describe details about the 7 surveyed villages, but they explained much details about the coverage in other villages in the Results, also the number of villages was also 7. I hope they could explain more about the methods and data they got from APS & INSD in the methods part. In addition, add some explanations as a footnote for the Table 3. In table 5, Can they add % in addition to the number?

**Author’ response**: Thank you for your comment. Firstly, as indicated in the previous question, Table 2 has been deleted and only Table 3 has been retained in the revised manuscript. It should be noted that table 3 do not present details of other villages, but of the municipalities in which our seven (7) villages are located. As mentioned in the "study area" section, the province is divided into municipalities and the municipalities into villages. Sissili is made up of 7 municipalities. We wanted to know the overall trend in the municipalities that make up the province before looking at the specific case of the villages in the municipalities. Since we could not survey all the villages in a commune, we decided to take an interest in the data on all the municipalities provided by the authorities and the NGO, and then verify this by interviewing the households in 7 villages ourselves.

Thus, for tables 2 and 3: information on the number of villages, population size and households was collected from the National Institute of Demographic Statistics (INSD), available in the population census report. Data on the number of latrines in existence in 2016 was collected from the Ministry in charge of sanitation and the NGO APS, which is the only structure to have implemented CLTS in the entire province. As before any CLTS project, a baseline situation concerning the number of latrines must be made by the implementing structure, in our case the NGO APS, so it appeared to be one of the ideal targets for the collection of certain data.

On the basis of Burkina Faso regulations, which stipulate that a latrine can only be used by 10 people, the authors calculated the number of latrines required to achieve a 100% coverage rate; then (by dividing the population size by 10), the latrine coverage rate in 2016 and also in 2020, based on the number of latrines recorded by the ministry in charge of sanitation and the NGO APS.

Now, to ensure the reliability of these results and the trends in latrine coverage, we have presented the results of surveys carried out by the authors in the 7 villages in Table 4, where the same information was sought. Apart from Table 2, which is a reconstitution of data collected from institutional and similar actors, the other Tables 3, 4 and 5 are the results of household surveys obtained by the authors.

As suggested by the reviewer, in addition to the numbers, the proportions (in %) have been added in all tables. Details of the calculation method have been added to the revised manuscript in the "methodology" section.

- In page 23, can they delete “the sad fact” since it is subjective expression.
  The all paragraphs starting from the “This low….” in page 24 should be moved to the discussion part since it is not result.

**Author’ response**: Thank you for your comment. The expression “*the sad fact*” has been removed. The paragraph “*this low....... that it should promote*” has been moved to the discussion section in the revised manuscript as requested.

- Discussion
  In the first paragraph of the discussion in page 26, “to some information” should be deleted or rephrased.

**Author’ response**: We thank the reviewer for this suggestion. The expression "to some information" in the first paragraph of the discussion on page 26 has been reworded. Thank you again for all your comments and time in reviewing our paper.

**Reviewer #2:**

- This manuscript offers important reinforcing evidence of the shortcomings of CLTS that have been raised elsewhere but which it does not cite (see for example: Trimmer, J et al. 2022. The Impact of Pro-Poor Sanitation Subsidies in Open Defecation-Free Communities: A Randomized, Controlled Trial in Rural Ghana. Environmental Health Perspectives. DOI:10.1289/EHP10443; Brown, J., et al. 2019. Community-Led Total Sanitation Moves the Needle on Ending Open Defecation in Zambia. American Journal of Tropical Medicine and Hygiene 100 (4) 767-769. DOI:10.4269/ajtmh.19-0151; and Cameron, L., et al. 2021 Sanitation, financial incentives and health spillovers: A cluster randomised trial. Journal of Health Economics. DOI:10.1016/j.jhealeco.2021.102456). Its interviews with households over two seasons along with field observations of the sanitation infrastructure are important, particularly as they lend themselves to the sobering recognition of the durability problem as perhaps the most critical shortcoming of CLTS. The authors’ questions about the effectiveness of CLTS’ zero-subsidy principles are also critical to understanding CLTS’ slippage issues.

**Author’ response**: Thank you for your comment and your support. The authors cited by the reviewer have been referenced in the revised manuscript.

- In its current state, however, this manuscript is unpublishable in a peer-reviewed venue. Most problematically, its stated objectives are inconsistent with the core scientific convention of posing neutral questions which are either answered (or not) by the evidence collected (see page 7 of the manuscript).

**Author’ response**: Thank you for your comment. A thorough reorganization of the original manuscript took place in order to make it publishable according to the journal's guides of authors. Also, the problematic formulation of the objectives which was also raised by the editor and the second has been solved in the revised manuscript. A new, more neutral formulation of the objective was made and put only in the introduction section of the revised manuscript.

- Determining that “CLTS increased coverage,” or that “the quality of latrines built under CLTS is poor” are statements of results, not neutral research objectives; indeed, framed as research objectives, they reveal bias. Instead, the objectives should be framed as questions, e.g. “how did CLTS change sanitation coverage in Sissili Province?” or “what is the failure rate of latrines installed under the CLTS program in Sissili Province?” or “what explains the high failure rate of latrines installed under CLTS in Sissili Province?”.

**Author’ response**: We thank the reviewer for this suggestion. We have taken them into account in reformulating the objectives in the revised manuscript. You can see the new presentation of objectives in the introduction section of the revised manuscript. Thank you again for this suggestion. Well appreciated.

- There are other significant issues. For example, the authors report two waves of data collection, but do not report results from each wave, nor do they do indicate how the two waves of data collection differed. From what I could tell, they report the two waves of household surveys as though it were a single cross-sectional survey collected during a single survey period, but it’s not clear.

**Author’ response**: Thank you for your comment. The study was carried out in two campaigns. The first in 2021 targeted only 3 villages in the Sissili province during the rainy season, and the second campaign, which took place the following year (2022) during the same period (rainy season), targeted the remaining 4 villages, giving a total of 7 villages. Given that the same questionnaire was administered to all households in 7 villages (in 2021 and 2022), it made no sense to present the results of 3 villages with the same variables and then present the results of the other 4 villages with the same variables. This explains why the survey results from 7 villages were combined.

Details have been added in the methodology section of the revised manuscript to enlighten the reader.

The reasons why we surveyed the 7 villages over 2 years were also given above, as one of the reviewers had requested them.

- Also: much of the results section presents data unrelated to the authors’ own household survey results. Tables 2 and 3 and Figure 8 all correspond to “corrected” self-reported latrine coverage data from the NGO APS.

**Author’ response**: Thank you for your comment. The data was collected not only from the NGO APS, but also from the Burkina Faso Ministry in charge of sanitation, the National Institute of Statistics and Demography (INSD) and households from 7 villages surveyed in the province of Sissili. The data in Table 2 came from these 3 institutions (NGO APS, Ministry of Sanitation and INSD), which were triangulated and gave the same answers. An error was made in the source of the table, as we omitted to add the INSD and the Ministry of Sanitation, which gave the impression that the data came solely from the NGO APS, whereas this was not the case, especially as the calculation of latrine coverage rates was done by the authors. In order to verify the data collected from these institutions, we carried out household surveys, the results of which are presented in Tables 4 and 5, and which confirmed the trends (increase in latrine coverage) shown in Table 3. Further details are given in the revised manuscript.

- Meanwhile, the objectives of the power calculations for the household surveys are not obvious to me. Typically power calcs are designed to communicate the smallest detectable difference between an intervention group and a counter-factual group with respect to a specific variable of interest in a controlled trial, or else, in the case of a quasi-experimental study, to explain the variation in a response variable with respect to the variation of one or more explanatory variables, or to determine the sample’s “representativeness” of a broader population. None of that is offered here. At best, this is an effort to collect some “ground truth” on CLTS performance in Sissili Province that may be illustrative of broader trends but not conclusive.

**Author’ response**: Thank you for your comment. The purpose of the household surveys was to find out how many households had at least one latrine (1), when these latrines were built (before or after the CLTS) for those households that did have them (2), what type of latrines were built under CLTS? (3), to know the resistance of these latrines built under CLTS (more precisely, to know if they have already collapsed since their construction, or if they are resistant to strong wind and rain) (4) and to know, in cases where there have already been collapses, at what period this generally occurred (5). All this was done to study the durability of latrines built under CLTS. It wasn't a study to explain a specific variable, which requires, as you said, an intervention group and a counterfactual (or control) group. On the basis of the results obtained, conclusions were drawn about the sustainability of latrines built under CLTS, and the contribution of CLTS to the evolution of the sanitation access rate.

- Also: the authors make claims about how CLTS has affected open defecation in Sissili province (they use the term “cessation of OD”) but they neither measure OD themselves nor refer to any other studies of open defecation (as distinct from latrine coverage or “access.”).

**Author’ response**: Thank you for your remark. The primary objective of the paper was not to assess the impact of CLTS on open defecation in the province, which explains why insufficient emphasis was placed on this aspect. However, we have taken your recommendation into account, but deleted the sentence in the revised manuscript. thank you again for your remark.

- Also: the recommendations regarding CLTS are intriguing, but they don’t necessarily follow directly from the data presented or directly collected by the authors, and are better suited for some kind of commentary or policy analysis piece, rather than a research article as presented here.

**Author’ response**: Thank you for your comment. This article is part of a doctoral thesis being carried out in Burkina Faso on the effectiveness and sustainability of CLTS in sub-Saharan African countries. The proposals made in the original manuscript were therefore part of a more global framework aimed at improving both the approach and the technologies implemented as part of CLTS, for greater sustainability. These proposals were addressed to the scientific community, implementation structures and politicians. For these reasons, this section gives the impression of a kind of political commentary or analysis.

However, as suggested by the reviewers and the editor and no longer part of our objectives in this paper, this section ("recommendations") has been removed in the revised version so as not to give our research paper a political connotation or make it look like a political document.

- In short, I very much hope that a sensible venue and format can be found for the team’s findings of latrine durability challenges and its associated drivers related to affordability of durable materials and professional latrine installation, but I don’t see an academic journal research article as offering them.

**Author’ response**: Thank you for your comment. Yes, this is indeed a thorny problem of the CLTS approach that we have tackled and provided some answers to, a contribution towards solving it. We hope that the revised version of the manuscript will boost research in this area. Thank you again for all your comments and time in reviewing our paper.
